# Infant Development at the Age of 6 Months in Relation to Feeding Practices, Iron Status, and Growth in a Peri-Urban Community of South Africa

**DOI:** 10.3390/nu10010073

**Published:** 2018-01-12

**Authors:** Marinel Rothman, Mieke Faber, Namukolo Covic, Tonderayi M. Matsungo, Marike Cockeran, Jane D. Kvalsvig, Cornelius M. Smuts

**Affiliations:** 1 Centre of Excellence for Nutrition (CEN), Faculty of Health Sciences, North-West University, Potchefstroom Campus, Private Bag x6001, Potchefstroom 2520, South Africa; marinel.rothman@gmail.com (M.R.); mieke.faber@mrc.ac.za (M.F.); N.Covic@cgiar.org (N.C.); tmatsungo@gmail.com (T.M.M.); 2 Non-Communicable Diseases Research Unit, South African Medical Research Council (SAMRC), PO Box 19070, Tygerberg 7505, South Africa; 3 Medicine Usage in South Africa (MUSA), North-West University, Potchefstroom Campus, Private Bag x6001, Potchefstroom 2520, South Africa; marike.cockeran@nwu.ac.za; 4 Department of Public Health Medicine, University of KwaZulu-Natal, Durban 4041, South Africa; kvalsvig@gmail.com

**Keywords:** infancy, psychomotor development, nutritional status

## Abstract

Background: Evidence on the association between feeding practices, iron deficiency, anaemia, stunting, and impaired psychomotor development during infancy is limited. This study assessed the association between psychomotor development with early feeding practices, growth, iron status, and anaemia. Methods: This was cross-sectional baseline data of a randomised controlled trial which included 6-month-old infants and their mothers or primary caregivers (*n* = 750) in a peri-urban community in the North West province of South Africa. The Kilifi Developmental Inventory and a parent rating scale were used to assess psychomotor development. Feeding practices and anthropometric measurements were based on the World Health Organisation (WHO) guidelines. Anaemia and iron status were determined by blood sample analysis. Results: Prevalence of anaemia and stunting for the infants were 36.4% and 28.5%, respectively. Multiple regression analysis showed that birth weight was related to combined psychomotor scores (*β* = −3.427 (−4.603, 1.891), *p* < 0.001), as well as parent rating scores (*β* = −0.843 (−1.507, −0.180), *p* = 0.013). Length-for-age *z*-scores were associated with combined psychomotor scores (*β* = −1.419 (−2.466, 0.373), *p* = 0.008), as well as parent rating scores (*β* = −0.747 (−1.483, −0.010), *p* = 0.047). Conclusions: In this setting, with high prevalence of anaemia and stunting, important associations between lower psychomotor development scores and birthweight as well as length-for-age *z*-scores in 6-month-old infants were found. These findings warrant further investigation to develop a greater understanding of factors influencing the association between child growth and psychomotor development within the first 1000 days of life.

## 1. Introduction

Infants and young children attain their optimal development through a combination of genetic potential, psychosocial stimulation, adequate nutrition, and a safe, clean physical environment [1]. An estimated 250 million children younger than 5 years (43%) in low- and middle-income countries, with 66% in Sub-Saharan Africa, are at risk of not reaching their development potential because of extreme poverty and stunting [2]. The first 1000 days of life (from conception to the child’s second birthday) is recognised as a critical period during which rapid growth and development occur [1]. Nutritional deficits during this period may cause long-term impairment in child growth and intellectual performance [3]. Optimal feeding practices, to ensure adequate energy and nutrient intake, are therefore of high priority.

Stunting and anaemia are two of the most common forms of undernutrition during early childhood, and both have been linked to delayed cognitive development [4]. Globally, 24.5% of children younger than 5 years have been reported to be stunted [5] and 47.4% anaemic [6,7], with iron deficiency (ID) being the primary cause of anaemia [8]. Iron deficiency is associated with an increased risk of morbidity in infants and young children, impaired physical and cognitive development, and reduced work productivity in adults [9]. In developing countries, infants are especially susceptible to iron deficiency because of the high amounts of iron required for their growth coupled with a diet low in bioavailable iron [10].

Various studies have reported on the cognitive development of infants and children in relation to anaemia and/or iron status. These studies showed an inverse association between iron status and fine motor skills in 9-month-old infants from China, Ghana, and USA [11]; low haemoglobin (Hb) concentrations (<95 g/L) at age 8 months were associated with impaired motor development at age 18 months [12], and infants with iron deficiency anaemia (IDA) had lower reaching and grasping patterns compared to non-IDA infants [13]. Several observational studies reported that children who were anaemic during infancy showed lower academic performance during their school-age years, even after treatment of the anaemia [14,15].

Poor nutrition is one of the main contributors to stunting and micronutrient deficiencies such as iron deficiency and, as a result, causes poor development [16]. The World Health Organisation (WHO) recommendations are that infants should be exclusively breastfed for the first 6 months of life [17]. However, a high percentage of infants are introduced to complementary foods before the age of 6 months in both high- and low-income countries, resulting in low exclusive breastfeeding rates of only 39%, according to the 2016 Global Nutrition Report [10]. Although globally exclusive breastfeeding rates are lowest in high-income countries, only 37% of infants under 6 months of age in low-income and middle-income countries are exclusively breastfed [18]. In South Africa, exclusive breastfeeding is low (32% of infants under 6 months of age in 2016) [19] and only 30% of children are breastfed until the age of 24 months [20]. Like many other African countries, in South Africa complementary foods often consist of cereal-based watery porridges with low nutritional quality [21,22], resulting in inadequate intakes of key micronutrients, such as iron, vitamin A, and zinc. This may affect the growth and development of the infants [3].

A birth cohort study reported that short duration of exclusive breastfeeding (≤2 months) was associated with poorer psychomotor development in 6–12-month-old Brazilian infants [23].

Baseline data of a recently completed randomised controlled trial (Clinicaltrials.gov NCT01845610) in South Africa showed that 28.5% of 6-month-old infants were stunted and 36.4% were anaemic. Short duration of exclusive breastfeeding (≤2 months) was reported for 48.9% of infants [24]. Against the background of a high prevalence of stunting and anaemia, as well as short duration of exclusive breastfeeding, we assessed whether stunting and anaemia were associated with early psychomotor development.

## 2. Methods

### 2.1. Study Population and Participants

Baseline data for 750 infants of age 6 months (5.85–7.01 months) who participated in a randomised controlled trial was collected during the period of September 2013 to January 2015. Study participants resided in the peri-urban Jouberton area in the greater Matlosana Municipality in Klerksdorp, North West province of South Africa. Jouberton is a low socioeconomic community 200 km from the nearest metropolitan area (Johannesburg). The number of study participants (*n* = 750) was based on sample size calculations for the randomised controlled trial, which had linear growth as the main outcome. Infants were excluded from the baseline for the randomised controlled trial if they had never been breastfed; suffered from severe obvious congenital abnormalities or a chronic disease; were severely anaemic (Hb < 70 g/L) or severely wasted - weight-for-length *z*-score < −3 SD(standard deviation) of the reference median; were known to be allergic or intolerant to peanuts, soy, milk, or lactose; received special nutritional supplements; were not a singleton, or if their mothers (primary caregivers) planned to move out of the study area within the next seven months. Infant-mother pairs were recruited through five primary health care clinics and house-to-house visits.

### 2.2. Ethical Standards and Disclosure

This study was conducted according to the Declaration of Helsinki guidelines. The study was approved by the Ethics Committee of the North-West University and the South African Medical Research Council. After institutional ethical approval was obtained, the study was reviewed by the Provincial Department of Health and Social Development for registration with the Directorate for Policy, Planning, and Research. Permission was also obtained at district and community levels to implement the study. Written informed consent was obtained from the mother or legal guardian. Infants who were found to be severely anaemic (Hb < 70 g/L) or severely wasted (weight-for-length *z*-score < −3 SD of the reference median) during screening were to be referred to a primary health care clinic and were excluded from the study.

Trial registration: The randomised control trial was registered as a clinical trial at the registry Clinicaltrials.gov (NCT01845610).

### 2.3. Data Collection

Data collection and informed consent were conducted at a central study site within the study area. The mother or primary caregiver (hereafter collectively referred to as caregivers) were interviewed in their preferred language by trained fieldworkers. Socioeconomic information on the household was collected using a structured questionnaire that has been used in similar settings [25].

Breastfeeding and complementary feeding practices were assessed retrospectively using a structured questionnaire based on WHO guidelines for assessing infant and young child feeding practices [26].

Information on the usual consumption of foods by the infants over the past seven days was collected using a set of unquantified food frequency questions that had previously been used in a similar study [25]. These questions were tested for face validity. The caregiver had a choice of four options to describe the child’s intake of listed foods. The four options were (i) every day; (ii) most days (not every day but at least four days during the past seven days); (iii) once a week (at least once, but less often than four days during the past seven days); and (iv) never.

Anaemia and iron status were determined by blood sample analysis. A professional nurse took the blood sample. In cases where blood was not obtained successfully via antecubital venepuncture of the arm area or dorsal area, a finger-prick blood sample was taken. A 4-mL blood sample was obtained from 485 infants and a finger-prick blood sample was obtained from 265 infants. Hb concentration was measured on all samples (*n* = 750) using the Hemocue that uses the direct cyanmethaemoglobin method. For infants from whom an antecubital venepuncture blood sample was obtained successfully (*n* = 485), after the Hb test was done, the remaining blood was centrifuged at 500× *g* for 15 min at room temperature and the plasma aliquots were stored at −80 °C.

Plasma ferritin (PF) concentration was determined using a sensitive Sandwich enzyme-linked immunosorbent assay (ELISA) technique [27]. High-sensitivity C-reactive protein (CRP) and alpha-1 glycoprotein (AGP) were measured with an ELISA kit from Human Diagnostics. The PF concentrations altered by inflammation were adjusted using correction factors (CFs), based on the elevated CRP and AGP status of the infant [28].

Anthropometric measurements were taken by two fieldworkers who were trained and standardised according to the WHO Training Course on Child Growth Assessment for infants [29]. Birth weight was recorded from the infant’s clinic growth monitoring card issued at birth.

We assessed, at the age of 6 months, psychomotor development based on direct testing and caregiver report, particularly with regard to two developmental domains, namely, locomotor development, which is related to the infant’s ability to select and modify his/her ongoing movement appropriately [30], and eye-hand coordination, which is the ability to reach or contact objects [31]. For direct testing, the Kilifi Developmental Inventory (KDI) has been developed to include culturally relevant items, and designed specifically for use in resource-poor settings by assessors with little experience in child development. It has been evaluated for reliability and validity in normal and disease-exposed populations in Kenya [32,33], and was recently used to study the effect of nutritional supplements on psychomotor development in infants and young children in Malawi [34], Ghana [35], and Kenya [36].

Caregiver report was based on the South African developed parent rating scale. The parent rating scale has previously been validated in a study that included children aged 6–36 months from rural and urban settings in South Africa [37]. In Kenya, developmental outcomes in children were assessed using both the KDI and parent rating scales, and a significant positive relationship between most KDI scores and parent rating scores was reported [32].

Both the KDI and the parent rating scale were translated into the local language by an experienced translator who had done this previously for two similar randomised controlled trials [38,39]. Verification of the translation was done during the training of the assessors and corrections where needed were made before final printing of the assessment tools. During the recruitment process for cognitive assessors, role play formed part of the interview process to identify suitable individuals to be trained. Two selected individuals were trained as cognitive assessors by an experienced assessor under the guidance of the study psychologist (J.D.K.). The training was done over a three-week period and included alternate cycles of observation, role play practice, and feedback using life size human infant dolls. During the final part of the training, each assessor had to complete the assessment for at least five infants who were not part of the study. Assessment scores were evaluated and an interrater reliability score of 90% had to be achieved between the assessors. The two assessors carried out the assessments in two separate rooms to avoid distracting the infants. The caregiver was asked to assist where needed so as to get the attention of the infant.

### 2.4. Processing and Statistical Analysis of the Data

Data on infant feeding practices were entered into an EpiInfo data base. Data obtained from socioeconomic background, anthropometric status, blood results, and child development were captured by ClinTech (India) International Pvt Ltd. (Bengaluru, India), which used Pharmaceutical Applications Release version 4.6.6. Data were analysed using SPSS for Windows, version 23 (SPSS Inc., Chicago, IL, USA).

Anaemia was defined as Hb < 110 g/L [40]; iron deficiency (ID) as PF < 12 μg/L; and iron deficiency anaemia (IDA) as both PF < 12 µg/L and Hb < 110 g/L [41].

The WHO growth standards were used to calculate age and gender-specific *z*-scores. Stunting was defined as a length-for-age *z*-score (LAZ) < −2SD, and overweight was defined as a weight-for-length *z*-score (WLZ) > 2SD. Low birth weight was defined as <2.5 kg [42].

KDI scores were calculated by adding up the scores recorded by the assessors (0 = unable to perform task, 1 = partially able to perform task, and 2 = able to perform task). For eye-hand coordination, some activities were scored on a scale of 0 (no level of the activity was performed successfully) to 4 (maximum levels of activity were performed successfully). The maximum possible score for eye-hand coordination was 27, and for loco-motor development it was 26. The combined psychomotor score was obtained by the sum of eye-hand and locomotor scores. Parent rating scores as recorded by the assessors were grouped based on, 0 = infant was not able, and 1 = infant was able. The adapted questionnaires for 6-month-old infants was based on 33 items, and thus a maximum score of 33 could be obtained.

Categorical variables are reported as frequencies and percentages. Continuous data were tested for normality using Shapiro-Wilk-test, and are reported as the mean with 95% confidence intervals (CI) for normally distributed data, or as the median with the interquartile range (IQR) for data not normally distributed. Independent samples *t*-tests were used to compare development scores between two groups such as gender. Three or more groups were compared using one-way analysis of variance (ANOVA); where ANOVA showed significant differences, post-hoc analyses were conducted with a Bonferroni correction. Effect sizes for psychomotor and parent rating scores were determined using Cohen’s *d* value to indicate the standardised difference between two means (0 = no practical significance; 0.2 = small practical significance; 0.5 = medium practical significance; 0.8 = large practical significance). Multiple linear regression analysis was used to investigate associations between developmental scores, feeding practices, and nutritional status. We used R-squared change to evaluate the importance of the predictor variables. Three models were created in sequence of (1) nutritional status such as haemoglobin and iron status, as well as anthropometric measurements; (2) nutritional status and feeding practices such as breastfeeding practices and frequencies of food intake; and (3) nutritional status, feeding practices, and demographical data such as gender and caregiver’s education level. For the multiple linear regression analysis, diagnostics plots of the residuals were performed to evaluate model adequacy in terms of normality, homogeneity of variance, and linearity. For all analyses, statistical significance was set at *p* < 0.05.

## 3. Results

Most (91.7%) of the primary caregivers interviewed were the mother of the infant. As indicated in Table 1, the mean (95% CI) age of the primary caregivers was 28.4 (27.8, 29.0) years; 80% had at least 10 years of formal education (grade 10 or above), and only 10.7% were married. The median (IQR) number of people living in the household was 5 (4, 7). In 27.6% of households no member earned a salary; while 87.4% of households were recipients of the child support grant (a social grant). Anaemia was prevalent in 36.4% of infants. The prevalence of ID and IDA was 16.1% and 10.5%, respectively. Stunting was prevalent in 28.5% of infants.

As shown in Table 2, female infants (*n* = 363) and those with caregivers who passed at least grade 10 (*n* = 601) scored higher for the parent rating scales (*p* = 0.002, *d* = 0.217; and *p* = 0.007, *d* = 0.227, respectively). Parent rating scales scored higher for infants who were exclusively breastfed for ≤2 months compared to infants who were exclusively breastfed up to the age of 5–6 months (*p* = 0.003; *d* = 0.167), as well as for infants who consumed commercial infant cereal frequently (*p* = <0.001, *d* = 0.430). Infants who were breastfed at age 6 months (*n* = 525), scored higher eye hand scores compared to those who were not breastfed at age 6 months (*p* = 0.013, *d* = 0.181). Stunted infants (*n* = 214) scored lower for combined psychomotor development and parent rating scores than non-stunted infants (*p* < 0.001; *d* = 0.322, *p* = 0.001; *d* = 0.229, respectively). Lower combined psychomotor development and parent rating scores were recorded for low birthweight infants (*p* = <0.001; *d* = 0.533 and *p* = 0.013; *d* = 0.265, respectively). No significant differences were observed for psychomotor scores and parent rating scores between households with at least one member earning a salary and those with no members earning a salary, as well as households receiving a child support grant and those not receiving a child support grant.

Results based on multiple linear regression analysis indicated that birth weight was negatively related to eye-hand (*β* = −1.893 (−2.759, −1.027), *p* < 0.001), locomotor (*β* = −1.354 (−2.058, −0.650), *p* < 0.001), and therefore also combined psychomotor scores (*β* = −3.427 (−4.603, 1.891), *p* < 0.001), as well as parent rating scores (*β* = −0.843 (−1.507, −0.180), *p* = 0.013). Length-for-age *z*-scores were negatively associated with combined psychomotor (*β* = −1.419 (−2.466, 0.373), *p* = 0.008), as well as parent rating scores (*β* = −0.747 (−1.483, −0.010), *p* = 0.047) (Table 3 and Table 4). Frequent consumption of infant cereal was positively related to parent rating scores (*β* = 1.376 (0.892, 1.860), *p* < 0.001). Parent rating scales scored lower for male subjects (*β* = 0.683 (0.238, 1.127), *p* = 0.003). Furthermore, compared to exclusive breastfeeding up to the age of 0–2 months, longer duration of exclusive breastfeeding was negatively related to parent rating scores; *β* = −0.715 (−1.209, −0.221), *p* = 0.005 for exclusive breastfeeding up to the age of 3–4 months; and *β* = −0.975 (−1.637, −0.314), *p* = 0.004 for exclusive breastfeeding up to the age of 5–6 months.

## 4. Discussion

It is important to investigate psychomotor development in the context wherein it occurs, especially when protective and risk factors are simultaneously present [43]. Even though several studies reported that psychomotor development is associated with nutritional status or breastfeeding practices [2,44,45,46,47], the association of psychomotor development at the age of 6 months (when WHO recommends the introduction of complementary feeding) with nutritional status, as well as early introduction of complementary foods (which were mostly commercial infant products in our study), has been largely unexplored.

Our study showed that differences in developmental scores were related to the level of education of the caregiver, gender on the infant, early feeding practices, and nutritional status.

It is well-known that stunting is among the major risk factors for failure to attain full developmental potential [48], which is also supported in our results. We found that stunted, as well as infants born with a low birthweight scored lower psychomotor scores. In the same study population, Matsungo et al. (2017) reported that birth weight (kg) was inversely associated with stunting [49], which may be a consequence of maternal short stature, combined with poor nutrition during pre-conception and pregnancy [50,51,52,53]. Victora et al. reported that, since low birthweight is more commonly found in infants whose mothers were stunted, the effect of stunting on cognition may be intergenerational [54]. Matsungo et al (2017) further reported that boys, who scored lower parent rating scores in our study, were 1.73 times (*p* = 0.017) more likely to be stunted than girls [49]. Higher stunting rates in boys concur with the findings from 16 demographic and health surveys from 10 sub-Saharan countries [55].

Delayed growth and long term effects on psychomotor development may be caused by anaemia and or iron deficiency [56]. We found that anaemia and iron-status were not associated with psychomotor development scores. According to a review paper by Jáuregui-Lobera [57], deficits in cognitive development were associated with ID, IDA, and non-IDA still needs to be explored and may appear at different ages; and even though Hb concentrations seem to correlate with cognitive performance, iron supplementation may improve cognitive functions regardless of the Hb concentration of those affected [57].

Nutritional status is often associated with early feeding practices such as breastfeeding and the quality of complementary feeding [15,58]. At age 6 months, 70.1% of infants in our study were still breastfeeding and 42.0% received formula milk feeds. Complementary foods most consumed were infant cereal (68.1%) and commercial jarred infant foods (22.7%), which were introduced mostly at 3–4 months of age [24]. Eye-hand coordination scores were higher for infants who were still being breastfed in comparison to those who were no longer being breastfed at age 6 months. Breast milk fat contains long chain polyunsaturated fatty acids (docosahexaenoic acid and arachidonic acid) which are important for the neurological development of a child [59]. Although we found that loco-motor scores were not significantly higher for breastfed infants, some beneficial effects of breastfeeding in the first 6 months may only become apparent at a later stage [46,47]. Frequent consumption of commercial jarred infant foods as well as commercial infant cereal were associated with higher parent rating scores.

A strength of this study is the large number of infants (*n* = 750) within a very narrow age bracket (6 months old), compared to the wider age range in studies that report on child development in relation to nutritional status [58,60]. A limitation of the study is that venous blood sample was not successfully obtained for all infants. As a result, PF values are available for a sub-sample only (*n* = 485) while Hb results are based on either finger prick or venous samples. We compared the Hb values obtained from capillary versus venous samples, and results showed that there was no significant difference (*p* = 0.657) between the two groups (unpublished data). Simmonds et al. concluded that in the absence of a venous sample, a capillary sample via finger prick can be used as a reliable alternative in field settings [61].

## 5. Conclusions

In conclusion, against the background of short duration of exclusive breastfeeding, early introduction of commercial infant products, and the prevalence of stunting, anaemia, and iron deficiency, multiple regression analysis showed associations in developmental scores. Our study’s results showed that psycho-motor development in 6-month-old infants was positively associated with length-for-age *z*-score and birth weight, as well as frequent consumption of commercial infant cereals. Further investigation to develop a greater understanding of the association between child growth and psychomotor development within the first 1000 days, as well as factors influencing this association and how best to address it, is warranted.

## Figures and Tables

**Table 1:** Socioeconomic characteristics of the primary caregivers and characteristics of the infants.

**Caregiver Characteristics (*n* = 750)**	
Mean (95% CI) age, years	28.4 (27.8, 29.0)
Percentage caregivers with at least grade 10 education	80.0
Percentage caregivers who were not married	89.3
**Household Characteristic (*n* = 750)**	
Median (IQR) number of people in household	5 (4,7)
Percentage of households with no person earning a salary	27.6
Percentage of households receiving a child support grant for at least one child	87.4
Percentage of households with access to a flush toilet	95.1
Percentage of households with access to tap inside the dwelling	29.3
Percentage of households with access to electricity in the dwelling	92.3
**Infant Characteristics (*n* = 750)**	
Percentage of male infants	51.6
Percentage of infants who were anaemic ^1^	36.4
Percentage of infants who were iron deficient ^2,3^	16.1
Percentage of infants with iron deficiency anaemia ^2,4^	10.5
Percentage of infants who were stunted ^5^	28.5
Percentage of infants who were overweight ^6^	10.1
Percentage of infants who had a low birth weight ^7^ (%)	14.0
**Psychomotor development scores (*n* = 750)**	
Mean (95% CI) combined psychomotor score (maximum possible score: 53)	36.8 (36.3, 37.2)
Mean (95% CI) eye-hand coordination sub-scale (maximum possible score: 27)	20.4 (20.1, 20.7)
Mean (95% CI) locomotor skills sub-scale (maximum possible score: 26)	16.3 (16.1, 16.6)
Mean (95% CI) Parent Rating score (maximum possible score: 33)	20.2 (19.9, 20.4)

CI = confidence interval; IQR = interquartile range. ^1^ Hb < 110 g/L; ^2^ *n* = 485; ^3^ PF < 12 µg/L, after adjustment for inflammation; ^4^ Hb < 110 g/L and PF < 12 µg/L, after adjustment for inflammation; ^5^ LAZ < −2SD; ^6^ WLZ > 2SD; ^7^ birth weight < 2.50 kg.

**Table 2:** Comparison between development scores and caregiver’s level of education, infant’s gender, early feeding practices, frequently consumed food items, anaemic and iron status, growth status, and birth weight of 6-month-old infants (mean, 95% CI).

		Eye-Hand (Max Score 27)			Locomotor (Max Score 26)			Combined Psychomotor (Max Score 53)			Parent Rating (Max Score 33)		
Variable	*n*	Mean, 95% CI	*p* ^1^	*d*	Mean, 95% CI	*p* ^1^	*d*	Mean, 95% CI	*p* ^1^	*d*	Mean, 95% CI	*p* ^1^	*d*
**Caregiver education**													
≥Grade 10	601	20.6 (20.2, 20.9)	0.078	0.159	16.5 (16.2, 16.8)	0.135	0.132	37.1 (36.6, 37.6)	0.059	2.806	20.3 (20.1, 20.6)	0.007	0.227
<Grade 10	138	19.93 (19.2, 20.6)			16.04 (15.5, 16.6)			35.9 (34.9, 37.0)			19.5 (18.9, 20.1)		
**Infant gender**													
Male	387	20.4 (20.0, 20.8)	0.938	0.005	16.2 (15.9, 16.5)	0.191	0.094	36.6 (36.0, 37.2)	0.530	0.046	19.8 (19.5, 20.1)	0.002	0.217
Female	363	20.4 (19.9, 20.8)			16.5 (16.2, 16.9)			36.9 (36.3, 37.6)			20.5 (20.2, 20.8)		
**Duration of EBF**													
Age 0–2 months	367	20.4 (19.9, 20.8)	0.735	0.025	16.5 (16.2, 16.8)	0.537	0.067	36.9 (36.3, 37.6)	0.711	0.021	20.5 (20.2, 20.8) ^2^	0.003 ^2^	0.167
Age 3–4 months	271	20.5 (20.0, 20.9)			16.3 (15.8, 16.7)			36.8 (35.9, 37.5)			19.9 (19.6, 20.4) ^2^		
Age 5–6 months	112	20.2 (19.4, 20.9)			16.2 (15.6, 16.8)			36.3 (35.2, 37.5)			19.4 (18.8, 20.1) ^2^		
**Breastfed at age 6 months**													
Yes	525	20.6 (20.3, 20.9)	0.013	0.181	16.4 (16.1, 16.7)	0.510	0.052	37.1 (36.5, 37.6)	0.058	0.147	20.2 (19.9, 20.5)	0.739	0.026
No	224	19.9 (19.3, 20.4)			16.3 (15.8, 16.7)			36.1 (35.2, 36.9)			20.1 (19.7, 20.5)		
**Foods consumed**													
**Formula milk**													
Frequent ^3^	311	20.2 (19.7, 20.7)	0.216	0.087	16.4 (16.0, 16.7)	0.998	0.000	36.6 (35.9, 37.3)	0.435	0.057	20.4 (20.1, 20.8)	0.069	0.132
Seldom/never	430	20.6 (20.2, 20.9)			16.4 (16.1, 16.7)			36.9 (36.3, 37.5)			20.0 (19.7, 20.3)		
**Commercial infant cereal**													
Frequent ^3^	503	20.5 (20.2, 20.9)	0.194	0.099	16.4 (16.1, 16.7)	0.728	0.026	36.9 (36.4, 37.5)	0.319	0.076	20.6 (20.4, 20.9)	<0.001	0.430
Seldom/never	236	20.1 (19.7, 20.6)			16.3 (15.9, 16.7)			36.4 (35.7, 37.2)			19.2 (18.8, 19.6)		
**Jarred infant foods**													
Frequent ^3^	168	20.5 (19.8, 21.2)	0.630	0.037	16.5 (16.1, 17.0)	0.490	0.059	37.1 (36.0, 38.1)	0.509	0.054	20.7 (20.3, 21.1)	0.014	0.212
Seldom/never	573	20.4 (20.1, 20.7)			16.3 (16.1, 16.6)			36.7 (36.2, 37.2)			20.0 (19.8, 20.3)		
**Anaemic status**													
Anaemic ^4^	274	20.7 (20.2, 21.2)	0.150	0.108	16.4 (16.0, 16.8)	0.777	0.021	37.1 (36.4, 37.8)	0.293	0.079	20.2 (19.8, 20.5)	0.948	0.004
Non-anaemic	476	20.2 (19.9, 20.6)			16.3 (16.1, 16.6)			36.6 (36.0, 37.2)			20.2 (19.9, 20.5)		
**ID-status**													
ID ^5^	78	20.8 (19.9, 21.7)	0.264	0.133	16.4 (15.6, 17.2)	0.879	0.018	37.2 (35.8, 38.7)	0.535	0.074	20.0 (19.4, 20.7)	0.690	0.049
Non-ID	407	20.3 (19.9, 20.7)			16.5 (16.2, 16.8)			36.8 (36.2, 37.4)			20.2 (19.9, 20.5)		
**IDA-status**													
IDA ^4,5^	51	21.2 (20.3, 22.2)	0.099	0.241	16.1 (15.1, 17.2)	0.431	0.101	37.4 (35.7, 39.0)	0.535	0.092	20.0 (19.2, 20.9)	0.803	0.037
Non-IDA	434	20.3 (19.9, 20.6)			16.5 (16.2, 16.8)			36.8 (36.2, 37.4)			20.2 (19.9, 20.5)		
**LAZ**													
Stunted ^6^	214	19.6 (19.0, 20.2)	0.001	0.259	15.6 (15.1, 16.1)	<0.001	0.313	35.2 (34.3, 36.1)	<0.001	0.322	19.6 (19.1, 20.1)	0.001	0.229
Non-stunted	536	20.7 (20.4, 21.0)			16.7 (16.4, 16.9)			37.4 (36.9, 37.9)			20.4 (20.2, 20.6)		
**WLZ**													
Overweight ^7^	76	20.9 (20.2, 21.6)	0.292	0.125	16.6 (15.9, 17.2)	0.552	0.071	37.4 (36.3, 38.6)	0.332	0.115	19.4 (18.7, 20.1)	0.027	0.267
Not-overweight	674	20.4 (20.0, 20.7)			16.3 (16.1, 16.6)			36.7 (36.2, 37.2)			20.2 (20.0, 20.5)		
**Birth weight**													
<2.50 kg	102	18.5 (17.6, 19.4)	<0.001	0.491	14.9 (14.2, 15.7)	<0.001	0.441	33.4 (31.9, 34.9)	<0.001	0.533	19.5 (18.8, 20.1)	0.013	0.265
≥2.50 kg	626	20.8 (20.5, 21.1)			16.7 (16.4, 16.9)			37.4 (36.9, 37.9)			20.3 (20.1, 20.6)		

EBF = Exclusive breastfeeding, CI = confidence interval. ^1^ Two groups: independent samples *t*-test; Three groups: one-way ANOVA; ^2^ Bonferroni post-hoc test indicated significant difference between 0–2 months and 5–6 months age groups (*p* = 0.005); ^3^ ≥4 days a week; ^4^ Hb < 110 g/L; ^5^ PF < 12 µg/L; ^6^ Length-for age *z*-score < −2; ^7^ Weight-for-length *z*-score > 2; *d* (Cohen’s *d*-value): 0 = no practical significance; 0.2 = small practical significance; 0.5 = medium practical significance; 0.8 = large practical significance.

**Table 3:** Multiple linear regression analysis for psychomotor development scores (*n* = 455).

	Eye-Hand Coordination			Locomotor			Combined Psychomotor		
Variable	*β* ^1^ (95% CI)	*β* ^2^	*p*	*β* ^1^ (95% CI)	*β* ^2^	*p*	*β* ^1^ (95% CI)	*β* ^2^	*p*
Length-for-age *z*-score	−0.645 (−1.314, 0.023)	−0.074	0.058	−0.774 (−1.317, −0.231)	−0.109	0.005	−1.419 (−2.466, 0.373)	−0.103	0.008
Weight-for-length *z*-score	0.496 (−0.466, 1.457)	0.038	0.312	0.184 (−0.597, 0.965)	0.017	0.644	0.680 (−0.826, 2.185)	0.033	0.376
Birth weight (kg)	−1.893 (−2.759, −1.027)	−0.166	<0.001	−1.354 (−2.058, −0.650)	−0.146	<0.001	−3.427 (−4.603, 1.891)	−0.180	<0.001
Not breastfeeding ^3^	−0.765 (−1.418, −0.111)	−0.087	0.022	−0.152 (−0.683, 0.379)	−0.021	0.575	−0.917 (−1.940, 0.107)	−0.066	0.079
*Duration of exclusive breastfeeding (age)*									
3–4 months ^4^	−0.036 (−0.681, 0.608)	−0.004	0.912	−0.240 (−0.764, 0.284)	−0.036	0.369	−0.276 (−1.286, 0.733)	−0.021	0.591
5–6 months ^4^	−0.511 (−1.375, 0.352)	−0.046	0.246	−0.448 (−1.150, 0.254)	−0.050	0.211	−0.959 (−2.311, 0.394)	−0.055	0.164
Frequent intake of jarred infant food ^5^	0.033 (−0.673, 0.740)	0.003	0.926	0.117 (−0.457, 0.691)	0.015	0.689	0.150 (−0.956, 1.257)	0.010	0.790
Frequent intake of commercial infant cereal ^5^	0.405 (−0.227, 1.037)	0.048	0.208	0.058 (−0.455, 0.572)	0.008	0.824	0.464 (−0.526, 1.453)	0.035	0.358
Caregiver passed at least grade 10 ^6^	0.482 (−0.261, 1.224)	0.048	0.203	0.347 (−0.257, 0.950)	0.042	0.260	0.828 (−0.334, 1.991)	0.052	0.162
Male infant ^7^	0.113 (−0.467, 0.693)	0.014	0.703	0.475 (0.003, 0.947)	0.074	0.049	0.588 (−0.321, 1.497)	0.047	0.205
Adjusted R^2^	0.045			0.041			0.057		

^1^ Unstandardised; ^2^ Standardised; Reference categories; ^3^ Currently breastfeeding; ^4^ Age 0–2 months; ^5^ Less than four days a week; ^6^ <Grade 10; ^7^ Female.

**Table 4:** Multiple linear regression analysis for parent rating score (*n* = 455).

	Parent Rating Score		
Variable	*β* ^1^ (95% CI)	*β* ^2^	*p*
Length-for age *z*-score	−0.416 (−0.928, 0.096)	−0.060	0.111
Weight-for-length *z*-score	−0.747 (−1.483, −0.010)	−0.072	0.047
Birth weight (kg)	−0.843 (−1.507, −0.180)	−0.094	0.013
Not breastfeeding ^3^	−0.284 (−0.785, 0.217)	−0.041	0.266
*Duration of exclusive breastfeeding (age)*			
3–4 months ^4^	−0.715 (−1.209, −0.221)	−0.110	0.005
5–6 months ^4^	−0.975 (−1.637, −0.314)	−0.112	0.004
Frequent intake of jarred infant food ^5^	0.419 (−0.122, 0.961)	0.056	0.129
Frequent intake of commercial infant cereal ^5^	1.376 (0.892, 1.860)	0.206	<0.001
Caregiver passed at least grade 10 ^6^	0.344 (−0.225, 0.913)	0.043	0.236
Male infant ^7^	0.683 (0.238, 1.127)	0.109	0.003
Adjusted R^2^	0.094		

^1^ Unstandardised; ^2^ Standardised; Reference categories; ^3^ Currently breastfeeding; ^4^ Age 0–2 months; ^5^ Less than four days a week; ^6^ <Grade 10; ^7^ Female.

## References

[1] Bentley M.E., Johnson S.L., Wasser H., Creed-Kanashiro H., Shroff M., Fernandez Rao S., Cunningham M. (2014). Formative research methods for designing culturally appropriate, integrated child nutrition and development interventions: An overview. Ann. N. Y. Acad. Sci..

[2] Black M.M., Walker S.P., Fernald L.C.H., Andersen C.T., DiGirolamo A.M., Lu C., McCoy D.C., Fink G., Shawar Y.R., Shiffman J. (2017). Early childhood coming of age. Science through the life-course. Lancet.

[3] Prado E.L., Dewey K.G. (2014). Nutrition and brain development in early life. Nutr. Rev..

[4] Frongillo E.A., Tofail F., Hamadani J.D., Warren A.M., Mehrin S.F. (2014). Measures and indicators for assessing impact of interventions integrating nutrition, health, and early childhood development. Ann. N. Y. Acad. Sci..

[5] World Health Organization (2015). World Health Statistics. http://apps.who.int/iris/bitstream/10665/170250/1/9789240694439_eng.pdf?ua=1&ua=1.

[6] Worldwide Prevalence of Anaemia 1993–2005. http://apps.who.int/iris/bitstream/10665/43894/1/9789241596657_eng.pdf.

[7] (2008). WHO Glob Database Anaemia. www.who.int/vmnis/anaemia/prevalence/summary/anaemia_data_status_t2/en/.

[8] World Health Organization Global Nutrition Targets 2025 Anaemia Policy Brief. Global Nutrition Targets 2025. http://www.who.int/nutrition/publications/globaltargets2025_policybrief_anaemia/en/.

[9] World Health Organization Micronutrient Deficiencies: Iron Deficiency Anaemia. http://www.who.int/nutrition/topics/ida/en/.

[10] International Food Policy Research Institute (2016). Global Nutrition Report 2016. Promise to Impact.

[11] Angulo-Barroso R.M., Schapiro L., Liang W., Rodriques O., Shafir T., Kaciroti N., Jacobson S.W., Lozoff B. (2011). Motor development in 9-month-old infants in relation to cultural differences and iron status. Dev. Psychobiol..

[12] Sherriff A., Emond A., Bell J.C., Golding J., ALSPAC Study Team (2001). Should infants be screened for anaemia? A prospective study investigating the relation between haemoglobin at 8, 12, and 18 months and development at 18 months. Arch. Dis. Child..

[13] Shafir T., Angulo-Barroso R., Jing Y., Angelilli M.L., Jacobson S.W., Lozoff B. (2008). Iron deficiency and infant motor development. Early Hum. Dev..

[14] Grantham-Mcgregor S.M., Ani C. (2001). A review of studies on the effect of iron deficiency on cognitive development in children. J. Nutr..

[15] Black M.M. (2003). Micronutrient deficiencies and cognitive functioning. J. Nutr..

[16] Daelmans B., Damstadt G.L., Lombardi J., Black M.M., Britto P.R., Lye S., Dua T., Bhutta Z.A., Richter L.M. (2017). Early childhood development: The foundation of sustainable development. Lancet.

[17] (2014). Global Nutrition Targets 2025: Breastfeeding Policy Brief. http://apps.who.int/iris/bitstream/10665/149022/1/WHO_NMH_NHD_14.7_eng.pdf.

[18] Victora C.G., Bahl R., Barros A.J.D., França G.V., Horton S., Krasevec J., Murch S., Sankar M.J., Walker N., Rollins N.C. (2016). Breastfeeding in the 21st century: Epidemiology, mechanisms, and lifelong effect. Lancet.

[19] South Africa Demographic and Health Survey 2016: Key Indicators. Https://dhsprogram.com/pubs/pdf/PR84/PR84.pdf.

[20] Shisana O., Labadarios D., Rehle T., Simbayi L., Zuma K., Dhansay A., Reddy P., Parker W., Hoosain E., Naidoo P. (2014). The South African National Health and Nutrition Examination Survey (SANHANES-1).

[21] Faber M., Laubscher R., Berti C. (2016). Poor dietary diversity and low nutrient density of the complementary diet for 6- to 24-month-old children in urban and rural KwaZulu-Natal, South Africa. Matern. Child Nutr..

[22] Du Plessis L., Kruger H., Sweet L. (2013). Complementary feeding: A critical window of opportunity from six months onwards. S. Afr. J. Clin. Nutr..

[23] Eickmann S.H., Malkes N.F.D.A., Lima M.D.C. (2012). Psychomotor development of preterm infants aged 6 to 12 months. São Paulo Med. J..

[24] Rothman A.M.P. (2015). Nutritional Status, Feeding Practices and Motor Development for 6-Month-Old Infants. Ph.D. Thesis.

[25] Smuts C.M., Dhansay M.A., Faber M., van Stuijvenberg M.E., Swanevelder S., Gross R., Benadé A.J.S. (2005). Efficacy of multiple micronutrient supplementation for improving anemia, micronutrient status, and growth in South African infants. J. Nutr..

[26] (2010). Indicators for Assessing Infant and Young Child Feeding Practices. Part 2: Measurement. http://apps.who.int/iris/bitstream/10665/44368/1/9789241599757_eng.pdf.

[27] Erhardt J.G., Estes J.E., Pfeiffer C.M., Biesalski H.K., Craft N.E. (2004). Combined measurement of ferritin, soluble transferrin receptor, retinol binding protein, and C-reactive protein by an inexpensive, sensitive, and simple sandwich enzyme-linked immunosorbent assay technique. J. Nutr..

[28] Thurnham D.I., Northrop-Clewes C.A., Knowles J. (2015). The use of adjustment factors to address the impact of inflammation on vitamin A and iron status in humans. J. Nutr..

[29] (2009). Training Course on Child Growth Assessment. http://www.who.int/childgrowth/training/module_h_directors_guide.pdf.

[30] Berger S.E., Adolph K.E. (2007). Learning and development in infant locomotion. Prog. Brain Res..

[31] Neggers Y., Goldenberg R.L., Ramey S.L., Cliver S.P. (2003). Maternal prepregnancy body mass index and psychomotor development in children. Acta Obstet. Gynecol. Scand..

[32] Abubakar A., Holding P., Van Baar A., Newton C.R.J.C., Van de Vijver F.J.R. (2008). Monitoring psychomotor development in a resource-limited setting: An evaluation of the Kilifi Developmental Inventory. Ann. Trop. Paediatr..

[33] Kitsao-Wekulo P., Holding P., Abubakar A., Kvalsvig J., Taylor H.G., King C.L. (2016). Describing normal development in an African setting: The utility of the Kilifi Developmental Inventory among young children at the Kenyan coast. Learn. Individ. Differ..

[34] Prado E.L., Phuka J., Maleta K., Ashorn P., Ashorn U., Vosti S.A., Dewey K.G. (2016). Provision of lipid-based nutrient supplements from age 6 to 18 months does not affect infant development scores in a randomized trial in Malawi. Matern. Child Health J..

[35] Prado E.L., Adu-Afarwuah S., Lartey A., Ocansey M., Ashorn P., Vosti S.A., Dewey K.G. (2016). Effects of pre- and post-natal lipid-based nutrient supplements on infant development in a randomized trial in Ghana. Early Hum. Dev..

[36] Kvalsvig J., Holding P., Taylor M., Chhagan M., Kitsao-Wekulo P., Zulu S., Mwangome F., Jaeggi T., Kauchali S., Brouwer I. (2013). Instapa WP6: The effects of micronutrient supplementation with reduced iron content on motor development in infants in a malarial area. Ann. Nutr. Metab..

[37] Kvalsvig J.D., Govender K., Taylor M. (2009). Research on the Age Validation of NELDS Related to the Cognitive Development of Children between 0 and 4 Years of Ages. http://www.education.gpg.gov.za/Documents/NELDSguidelines8Jan.pdf.

[38] Taljaard C., Covic N.M., van Graan A.E., Kruger H.S., Smuts C.M., Baumgartner J., Kvalsvig J.D., Wright H.H., van Stuijvenberg M.E., Jerling J. (2013). Effects of a multi-micronutrient-fortified beverage, with and without sugar, on growth and cognition in South African schoolchildren: A randomised, double-blind, controlled intervention. Br. J. Nutr..

[39] Ogunlade A.O., Kruger H.S., Jerling J.C., Smuts C.M., Covic N., Hanekom S.M., Mamabolo R.L., Kvalsvig J. (2011). Point-of-use micronutrient fortification: Lessons learned in implementing a preschool-based pilot trial in South Africa. Int. J. Food Sci. Nutr..

[40] Haemoglobin Concentrations for the Diagnosis of Anaemia and Assessment of Severity. Vitamin and Mineral Nutrition Information System 2011. http://www.who.int/vmnis/indicators/haemoglobin.pdf.

[41] (2011). Serum Ferritin Concentrations for the Assessment of Iron Status and Iron Deficiency in Populations. Vitamin and Mineral Nutrition Information System. http://www.who.int/vmnis/indicators/serum_ferritin.pdf.

[42] Wang Y., Chen H.J., Preedy V.R. (2012). Use of Percentiles and *Z*-Scores in Anthropometry. Handbook of Anthropometry.

[43] (2009). Examining Early Child Development in Low-Income Countries. http://siteresources.worldbank.org/EXTAFRREGTOPEDUCATION/Resources/444707-1291071725351/ExaminingECDtoolkitFULL.pdf.

[44] Luo R., Shi Y., Zhou H., Yue A., Zhang L., Sylvia S., Medina A., Rozelle S. (2015). Micronutrient deficiencies and developmental delays among infants: Evidence from a cross-sectional survey in rural China. BMJ Open.

[45] Shafir T., Angulo-Barroso R., Su J., Jacobson S.W., Lozoff B. (2009). Iron deficiency anemia in infancy and reach and grasp development. Infant Behav. Dev..

[46] McCrory C., Murray A. (2013). The effect of breastfeeding on neuro-development in infancy. Matern. Child Health J..

[47] Gómez-Sanchiz M., Cañete R., Rodero I., Baeza J.E., Acila O. (2003). Influence of breast-feeding on mental and psychomotor development. Clin. Pediatr. Phila.

[48] Prendergast A.J., Humphrey J.H. (2014). The stunting syndrome in developing countries. Paediatr. Int. Child Health.

[49] Matsungo T.M., Kruger H.S., Faber M., Rothman M., Smuts C.M. (2017). The prevalence and factors associated with stunting among infants aged 6 months in a peri-urban South African community. Public Health Nutr..

[50] Black R.E., Victora C.G., Walker S.P., Bhutta Z.A., Christian P., de Onis M., Ezzati M., Grantham-McGregor S., Katz J., Martorell R. (2013). Maternal and child undernutrition and overweight in low-income and middle-income countries. Lancet.

[51] Gernand A.D., Christian P., Paul R.R., Shaikh S., Labrique A.B., Schulze K.J., Shamim A.A., West K.P. (2012). Maternal weight and body composition during pregnancy are associated with placental and birth weight in rural Bangladesh. J. Nutr..

[52] Catov J.M., Bodnar L.M., Olsen J., Olsen S., Nohr E. (2011). Periconceptional multivitamin use and risk of preterm or small-for-gestational-age births in the Danish National Birth Cohort. Am. J. Clin. Nutr..

[53] Owens S., Gulati R., Fulford A.J., Sosseh F., Denison F.C., Brabin B.J., Prentice A.M. (2015). Periconceptional multiple-micronutrient supplementation and placental function in rural Gambian women: A double-blind, randomized, placebo-controlled trial. Am. J. Clin. Nutr..

[54] Victora C.G., Adair L., Fall C., Hallal P.C., Martorell R., Richter L., Sachdev H.S. (2008). Maternal and child undernutrition: Consequences for adult health and human capital. Lancet.

[55] Wamani H., Åstrøm A.N., Peterson S., Tumwine J.K., Tylleskär T. (2007). Boys are more stunted than girls in sub-Saharan Africa: A meta-analysis of 16 demographic and health surveys. BMC Pediatr..

[56] Lozoff B. (2007). Iron deficiency and child development. Food Nutr. Bull..

[57] Jáuregui-Lobera I. (2014). Iron deficiency and cognitive functions. Neuropsychiatr. Dis. Treat..

[58] Grantham-Mcgregor S.M., Fernald L.C.H., Kagawa R.M., Walker S. (2014). Effects of integrated child development and nutrition interventions on child development and nutritional status. Ann. N. Y. Acad. Sci..

[59] World Health Organization (2009). Infant and Young Child. Feeding: Model. Chapter for Textbooks for Medical Students and Allied Health Professionals.

[60] Beard J.L. (2008). Why iron deficiency is important in infant development. J. Nutr..

[61] Simmonds M.J., Baskurt O.K., Meiselman H.J., Marshall-Gradisnik S.M. (2011). A comparison of capillary and venous blood sampling methods for the use in haemorheology studies. Clin. Hemorheol. Microcirc..

